# Parental perception of listening difficulties: an interaction between weaknesses in language processing and ability to sustain attention

**DOI:** 10.1038/s41598-018-25316-9

**Published:** 2018-05-03

**Authors:** Hettie Roebuck, Johanna G. Barry

**Affiliations:** 1 MRC Institute of Hearing Research, Division of Clinical Neuroscience, School of Medicine, University of Nottingham, Nottingham, United Kingdom; 2 Nottingham University Hospital NHS Trust, Queen’s Medical Centre, Nottingham, United Kingdom; 3 Department of Psychology, University of Wisconsin, Madison, USA

## Abstract

(Central) auditory processing disorder ((C)APD) is a controversial diagnostic category which may be an artefact of referral route. Yet referral route must, to some extent, be influenced by a child’s profile of presenting symptoms. This study tested the hypothesis that parental perception of listening difficulty is associated with weaknesses in ability to sustain attention while listening to speech. Forty-four children (24 with listening difficulties) detected targets embedded in a 16-minute story. The targets were either mispronunciations or nonsense words. Sentence context was modulated to separate out effects due to deficits in language processing from effects due to deficits in attention. Children with listening difficulties missed more targets than children with typical listening abilities. Both groups of children were initially sensitive to sentence context, but this declined over time in the children with listening difficulties. A report-based measure of language abilities captured the majority of variance in a measure capturing time-related changes in sensitivity to context. Overall, the findings suggest parents perceive children to have listening, not language difficulties, because weaknesses in language processing only emerge when stressed by the additional demands associated with attending to, and processing, speech over extended periods of time.

## Introduction

Children referred for suspected (Central) Auditory Processing Disorder (hence, APD) present with normal hearing, but have difficulty understanding speech, particularly when there is background noise. As originally conceptualised, the disorder was specific to the processing of auditory inputs^1,2^. However, the status of APD as a valid diagnostic category is controversial^3,4^. Most children suspected of being affected by the disorder also have symptoms commonly associated with specific language impairment (SLI) or dyslexia^5^, such as: poor working memory, delays in language and/or reading and poor attention. Individual children also vary in the extent to which these symptoms manifest.

To date, it has proven impossible to unambiguously attribute symptoms associated with APD to processing difficulties within the auditory system^6^. It has also proven difficult to distinguish between children diagnosed with APD and children diagnosed with difficulties in either language^7,8^, or reading^9^. As a consequence, it has been suggested that APD may be an artefact of referral route^8,10^. Yet, the fact remains, children who are objectively indistinguishable on standardised measures of listening or language follow different referral routes and receive different diagnoses. This suggests differences in key aspects of a child’s profile of presenting symptoms influence parental/clinical decisions about the most appropriate referral route to follow^11^.

There is great interest in understanding more about how disorders differ in presenting symptoms. Questionnaires can potentially provide insight into this issue^11,12^, but these measures also need to be supplemented by behavioural measures. Here, we combine behavioural and report-based measures to investigate the relationship between ability to sustain attention and symptoms of listening difficulty.

## APD and Attention

Symptoms of inattention, which are more commonly associated with attention deficit hyperactivity disorder (ADHD), have long been noted in APD^13^, but until recently, any contribution from cognitive influences, like attention or memory, to the difficulties associated with APD have been explicitly excluded by definition^1,2^.

Views regarding the nature of APD changed following a population-based study undertaken by Moore and colleagues^6^. The study was designed to identify the most reliable and informative combination of auditory tasks required for unambiguously diagnosing the disorder^14^. However, despite including the best available candidate tests of auditory processing abilities, the study failed to demonstrate a reliable link between measured thresholds of auditory perception and symptoms of listening, language or reading difficulty. Instead, these symptoms were found to associate more reliably with response inconsistency during testing and the authors hypothesised that APD was a disorder of attention.

The population-based study^6^ proved seminal in changing views about the nature of APD^15^. It is now accepted that a complex array of cognitive abilities contributes to difficulties in auditory processing^12,16,17^. To explicitly acknowledge this, affected children are now, as here, frequently referred to as having listening difficulties (LiD)^18,19^.

The hypothesis that APD was a disorder of attention^6^ was based on response inconsistency observed when children were doing psychophysical tasks largely involving non-speech stimuli. It is not clear whether this response inconsistency translates into symptoms of inattention, nor it is clear whether, or how, these symptoms contribute to the perception that a child has listening difficulties.

To our knowledge, only a few studies have specifically assessed sustained attention in APD^17,20^. In all cases, they have used some form of continuous performance task (CPT)^21^. In these tasks, participants are required to attend to streams of simple stimuli (e.g., a seen or heard ‘1’ and ‘2’), responding to infrequent targets (e.g., ‘1’) and ignoring frequent non-targets (e.g. ‘2’). While studies using CPTs demonstrate weaknesses in attention in children referred for APD, the findings do not provide compelling evidence that the deficits are central to the disorder.

The CPT, however, is an artificial task and interpretations may be confounded by stimulus specific effects^22^. It provides insight into the ability to maintain a level of on-task focus, but it is not designed to capture the complex range of cognitive processes that are additionally engaged while listening to connected speech over extended periods of time^23^.

## Probing lapses in attention while listening

To address our study interests, we required a task that involved listening to a passage of speech for an extended period of time. The task needed to offer some means of probing for lapses in attention. It also needed to offer some means of probing the impact of deficits in language processing on performance, since there is some controversy about the relationship between APD and SLI^7^ and problems sustaining attention have also been noted in children with SLI^24^.

A task developed by Cole and Perfetti^25^, for use in children as young as five years of age up to undergraduate level, satisified all our study requirements. It involved listening to a simple story and detecting mispronunciations embedded within it. Though not of specific interest for Cole and Perfetti, the task involved a high level of focused attention whilst detecting targets over an extended period of time. Since story tasks have been used to assess the capacity to sustain attention in a more ecological listening situation than standard CPTs^23^, it seemed a suitable task for adapting to address our research interests.

Cole and Perfetti’s task was originally developed to assess the role of sentence context in supporting word recognition in young children. To do this, Cole and Perfetti manipulated the sentence context preceding the words on which the mispronunciations were based and showed how both children and adults were faster and more accurate at detecting mispronunciations when based on words that could be predicted from the preceding context.

In addition to the mispronunciations, we incorporated nonsense words. These, we reasoned, would offer context-free probes for capturing lapses in sustained attention, thus providing us with an additional means for partialling out effects due to language difficulties, from those due to attention.

## Study hypothesis and predictions

The primary hypothesis for the study was that parents associate lapses in attention with difficulties in listening. Consistent with this hypothesis, we made the following predictions:

1. Children rated by parents as having listening difficulties (LiD) will miss more targets (nonsense and mispronunciation) embedded in the continuous listening task, than children rated as having typical listening abilities (TLi).
2. Regardless of listening ability, all children will detect predictable targets more reliably and more quickly than unpredictable targets.
  Attentional availability declines with long periods of listening^23^ and, reflecting the design of our task, we further predicted:
3. Numbers of targets missed will increase from the first, to the second half of the task.

Two target types were of particular interest for understanding impact of time on ability to sustain attention: nonsense words and predictable mispronunciations.

Detection of predictable targets offers insight into the effect on language processing of time-on-task, while nonsense target misses should reflect lapses in attention, since detection will not be influenced by sentence context or priming due to prior exposure.

Given the close interrelationship between listening, language and attention, it could also be that listening difficulties primarily reflect underlying language difficulties. If this were the case, the children with listening difficulties would not be predicted to demonstrate a sensitivity to sentence context.

## Results

### Word recognition and target identification

Regardless of listening status (LiD or TLi), all participants correctly matched all target words with the corresponding picture/feature in the word recognition task, confirming that the vocabulary used in the continuous listening task was appropriate for both groups of children.

Six participants, 1 TLi and 5 LiD, missed the same targets in both the target identification and continuous listening tasks. The numbers of targets missed in both tasks by each participant, ranged from between 1–6 targets. These targets were excluded and appropriately accounted for when determining the percent targets missed for these children in the continuous listening task.

Finally, both groups of participants provided correct responses to eight questions about the story (TLi: M = 7.4 SD = 1.0; LiD: M = 6.7 SD = 1.2), confirming they had listened to it, while doing the primary detection task.

### Association between listening difficulties and lapses of attention

To assess our primary hypothesis, we compared numbers of missed targets and reaction times for the children with LiD versus those with TLi. Most of the children with LiD had been referred for clinical assessment by an audiologist (LiD-Ref), but a substantial minority (n = 7; LiD-NonRef) had not. Report-based measures suggested the group was indistinguishable from the LiD-Ref group (Table 1), but for this first analysis they were analysed as distinct sub-group with LiD, since it was not clear if the nature of their listening difficulties was the same as those of the clinically-referred group.

**Table 1:** Summary of the participants grouped according to listening ability, together with their respective scores on key behavioural and parental report measures (Mean (SD), Range).

Group	TLi (n = 20)	LiD-NonRef (n = 7)	LiD-Ref (n = 17)	F-ratio, p value Group Comparison
Age (years)	9.8 (1.3)	9.3 (1.1)	9.7 (1.6)	n.s.
	8.0–12.8	8.0–10.8	7.0–13.0	
Gender (F:M)	08:12	03:07	09:08	
NVIQ (T-Score)	108.5 (12.0)	98.7 (10.5)	103 (13.0)	n.s.
	83–123	86–117	83–127	
Digit Span Back (SS)	10.5 (3.2)	9.1 (2.5)	9.8 (2.6)	n.s.
	7–15	7–13	5–16	
ECLiPS: SAP (SS)	9.8 (2.6)	4.3 (1.8)	2.3 (2.6)	F(1, 40) 63.98, p < 0.001
	7–14	1–6	1–10	TLi > LiD-NonRef ~LiD-Ref
ECLiPS: LLL (SS)	9.5 (3.1)	4.9 (3.0)	3.4 (3.0)	F(1, 40) 29.14, p < 0.001
	3–14	2–9	0–10	TLi > LiD-NonRef ~LiD-Ref
CCC-2: GCC	84.6 (16.0)	51.4 (21.7)	44.4 (18.5)	F(1, 40) 46.32, p < 0.001
(SS composite)	51–104	25–86	14–85	TLi > LiD-NonRef ~LiD-Ref
CPRS-R:S	49.7 (5.4)	66.3 (16.0)	60.4 (11.7)	F(1, 40) 17.56, p < 0.001
Cog. Probs/Inattn. (T-Score)	42–59	44–84	46–79	TLi < LiD-NonRef ~LiD-Ref

n.s. = non-significant.

ECLiPS: SAP = *Speech & Auditory Processing*; LLL = *Language/Literacy/Laterality*.

CCC-2: GCC = General Communication Composite.

Conners’ PRS-R:S Cog. Probs/Inattn. = Cognitive problems/Inattention.

**Figure 1:**
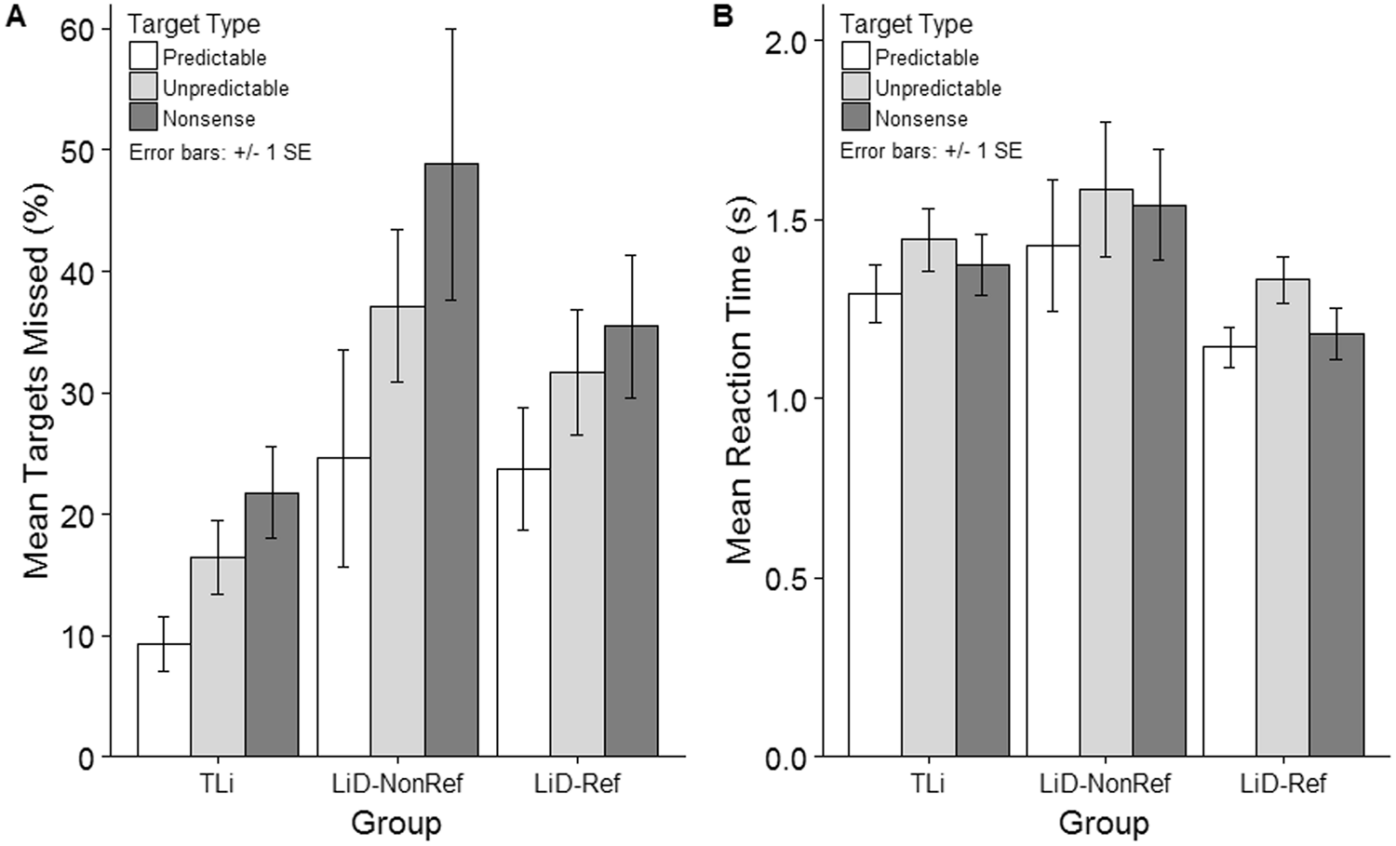
Effect of context on Percentage Targets Missed (panel A) and Reaction Time (seconds) (panel B) for the children with normal listening abilities (TLi) and the children with listening difficulties (Li-Ref; Li-Nonref). Error bars refer to standard errors.

Propensity for missed targets was compared using a mixed repeated measures ANOVA (Target Type [Predictable, Unpredictable, Nonsense] x Group [TLi, LiD-Ref, LiD-NonRef]) (Fig. 1).

There was a main effect of Target Type (F(1.71, 70.15) = 39.81, p < 0.001, η^2^ = 0.49). Participants missed more unpredictable (M = 28.43%, SD = 19.29) than predictable targets (M = 19.21%, SD = 19.16, p < 0.001). However, they also missed more nonsense targets (M = 35.36%, SD = 24.53), than predictable (p < 0.001), or unpredictable (p = 0.002) targets.

In support of our first two predictions, there was a main effect for Group (F(2, 41) = 5.29, p = 0.009, η^2^ = 0.21), but no Target Type x Group interaction (F(3.42, 70.15) = 1.27, p = 0.29, η^2^ = 0.06). Both LiD groups (LiD-Ref [M = 30.29%, SD = 17.85, p = 0.043] and LiD-NonRef [M = 36.86%, SD = 17.86, p = 0.025]) missed more targets, than the TLi group (M = 15.85%, SD = 17.85). There was no difference in performance between the two LiD groups (p = 1.00).

Effect of context on reaction time was also assessed using a mixed repeated-measures ANOVA (Target Type [Predictable, Unpredictable, Nonsense] x Group [TLi, LiD-Ref, LiD-NonRef]). There was a significant main effect for Target Type (F(2, 82) = 19.32, p < 0.001, η^2^ = 0.32). Predictable targets were detected more quickly (M = 1287 ms, SD = 371.28) than either the unpredictable (M = 1452 ms, SD = 411.26, p < 0.001), or the nonsense targets (M = 1364 ms, SD = 391.17, p = 0.017). Nonsense targets were detected more quickly than unpredictable targets (p = 0.018). There was no significant difference in reaction times between groups (F(2, 41) = 2.11, p = 0.14, η^2^ = 0.01), or any Target x Group interaction (F(4, 82) = 0.91, p = 0.46, η^2^ = 0.09). This pattern of results provides further evidence in support of prediction 2. Children with LiD are able to benefit from sentence context, despite missing more targets than children with TLi.

Despite not having a clinical referral for listening difficulties, the LiD-NonRef group was indistinguishable from the LiD-Ref group on the continuous listening task, suggesting the nature of their difficulties was similar. The two groups were therefore combined for all further analyses.

### Impact of time-on-task on target detection

To explore the impact of time-on-task on attention and language processing (prediction 3), we exploited an implicit subdivision in the continuous listening task. Percentage missed targets for predictable (high context effects) and nonsense targets (no context effects) were compared between the first and second halves of the task (Half [1, 2] × Target Type [Predictable, Nonsense] × Group [TLi, LiD]).

**Figure 2:**
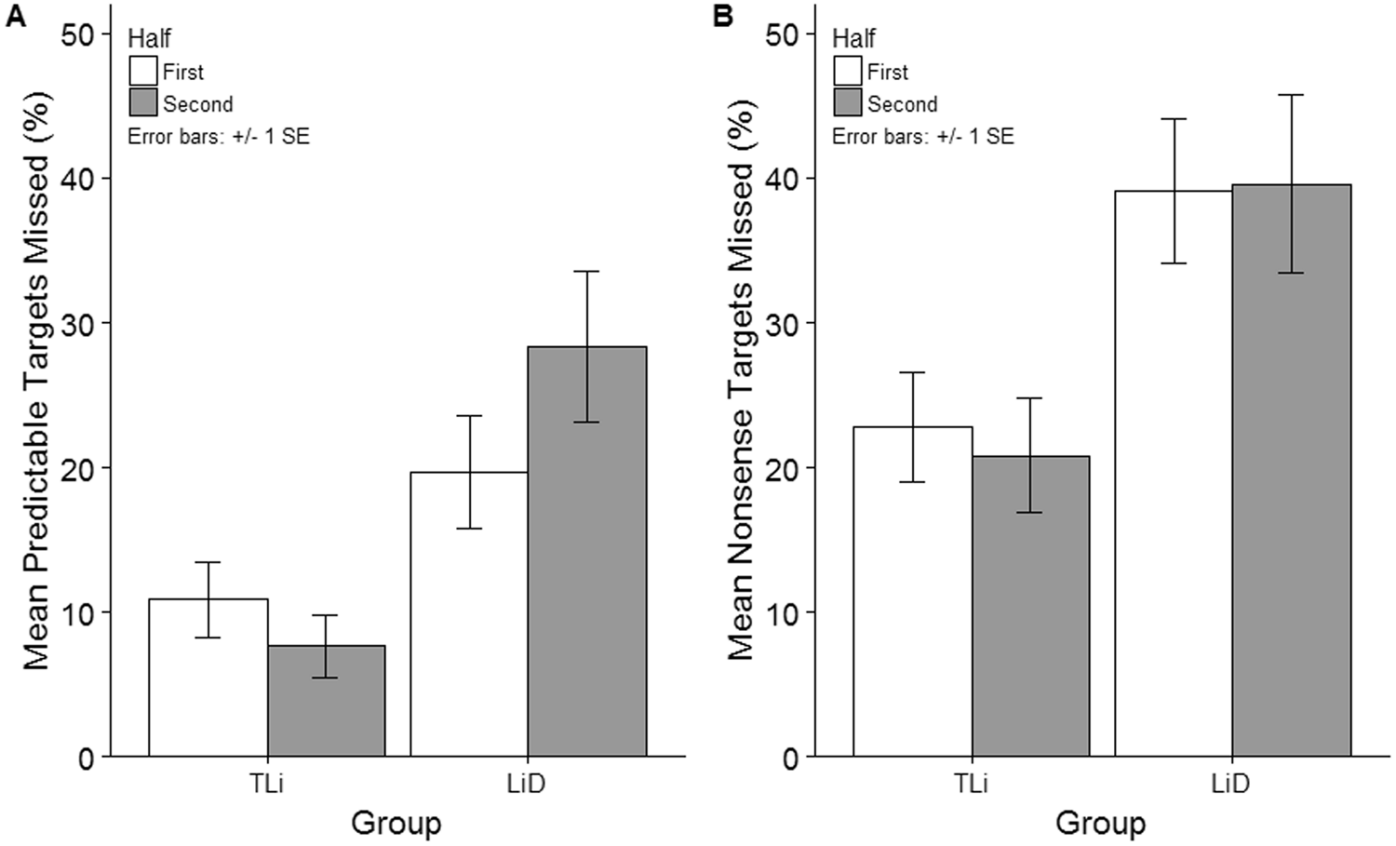
Effect of time-on-task according to Target Type [Predictable (panel A), Nonsense (panel B)], Half [1^st^ Half, 2^nd^ Half] × Group [TLi, LiD]. Error bars refer to standard errors.

There was no main effect of Half (F(1, 42) = 0.01, p = 0.93, η^2^ < 0.01), but there was a significant Half x Group interaction (F(1, 42) = 4.19, p = 0.047, η^2^ = 0.09), and a significant Half × Target Type × Group interaction (F(1, 42) = 5.56, p = 0.023, η^2^ = 0.18) (Fig. 2).

The LiD group missed more predictable words in the second half of the task compared with the first (Half 1: M = 19.66%, SD = 16.12 versus Half 2: M = 28.29%, SD = 20.04, p = 0.011). By contrast, time-on-task did not impact the performance of the TLi group (Half 1: M = 10.87%, SD = 16.05 versus Half 2: M = 7.64%, SD = 20.04, p = 0.16).

There was no significant difference in detection of nonsense targets with time-on-task in either the LiD group (Half 1: M = 39.12%, SD = 21.51 versus Half 2: M = 39.57%, SD = 25.18, p = 0.69) or the TLi group (Half 1: M = 22.77%, SD = 21.42 versus Half 2: M = 20.82%, SD = 25.18, p = 0.34).

In summary, detection misses increased with time-on-task in the LiD group only. The effect was limited to changes in sensitivity to sentence context, and was not observed for the nonsense targets.

### Factors influencing time-specific changes in sensitivity to sentence context

To further explore the factors influencing changes in sensitivity to sentence context, a derived measure (Context Sensitivity Change) was developed based on a subtraction of predictable target misses (Half-1 – Half-2). Context Sensitivity Change scores >0 indicate fewer targets missed with time-on-task, while scores <0 indicate more targets missed with time-on-task.

To better understand the factors contributing to changes in target detection over time, correlations (Table 2) were performed between Context Sensitivity Change scores, Age, NVIQ, Working memory (digit span backwards), Attention (Conners’: Cognitive problems/Inattention), Listening (ECLiPS: SAP) and Language (CCC-2: GCC).

**Table 2:** Correlations for the Total misses and Context Sensitivity Change scores with Age, NVIQ, Working memory (digit span backwards), Attention, Listening (SAP), and Language (GCC). (Pearson’s 2-tailed tests, corrections Holm-Bonferroni, p-value < 0.05 bolded).

	Total misses (r, p-value)	Context Sensitivity Change Predict (Half 1-Half2) (r, p-value)
Age (years) (n = 44)	−**0.568, 0.001**	0.068, 0.661
NVIQ (T-Score) (n = 44)	−0.292, 0.054	0.363, 0.015
Working memory (SS) (n = 44)	−0.375, 0.012	0.246, 0.107
Attention (T-score) (n = 41)	0.316, 0.044	−**0.506, 0.001**
Listening (SS) (n = 41)	−0.411, 0.008	**0.466, 0.002**
Language (SS composite) (n = 41)	−0.405, 0.009	**0.608, 0.001**

Listening and Attention were expected to have an increasing influence on performance over time and were therefore predicted to correlate with Context Sensitivity Change scores. Age, Working memory, NVIQ and Language were expected to exert a consistent influence over time and hence not predicted to correlate with Context Sensitivity Change scores.

To assess which factors contributed to task performance more generally, we performed the same correlations with Total Target misses.

Effects due to Attention and Listening correlated with Context Sensitivity Change scores (Table 2). Children rated by parents as having poor attention or listening skills got worse over time. Pre-existing weaknesses in Language, which were predicted to have a consistent influence on performance over time, also correlated with the Context Sensitivity Change scores.

Working Memory and Age associated with Total Target misses, though only Age remained statistically significant after correction for multiple comparison. By contrast, neither variable associated with changes in sensitivity to context over time.

### Influence of Language, Listening and Attention on performance over time

Language, Listening and Attention were entered into a stepwise linear regression with Context Sensitivity Change scores as the dependent variable, and using a probability of F < 0.05 as the criterion for variable entry and probability of F > 0.1 as the criterion for variable removal.

**Figure 3:**
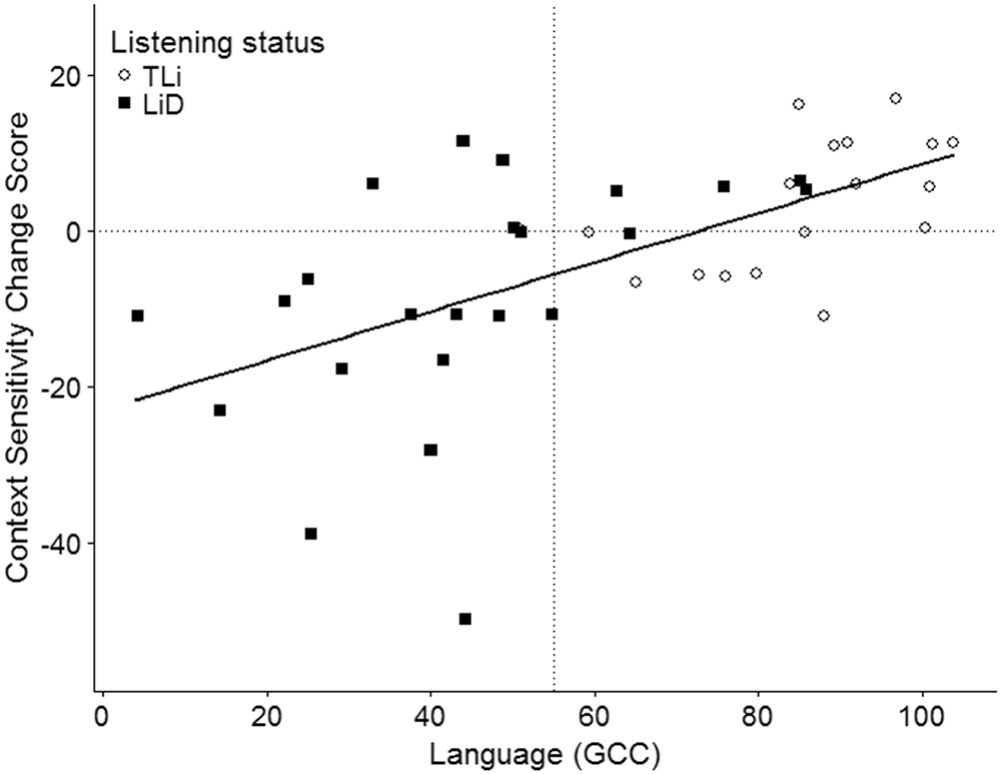
Correlation between the Context Sensitivity Change scores, and Language (GCC). GCC <55 indicates significant language difficulty (vertical dotted line). Context Sensitivity Change scores <0 indicate increasingly poor target detection over time.

Language explained 38% of the variance (R^2^ = 0.38, F(1, 38) = 22.36, β = 0.42, p < 0.001) (Fig. 3). However, Listening and Language, in particular, correlate highly (r = 0.83) causing problems with collinearity within the analysis. To further assess how much, or whether Listening, Language and Attention individually explain variance in Context Sensitivity Change Scores, a series of partial correlations were performed with each variable, while controlling for the influence of the other two (Table 3). Consistent with the regression analysis, neither Attention nor Listening explained significant variance in the Context Sensitivity Change scores, after contributions from Language were partialled out. Language remained significant, after contributions from the two other variables were partialled out.

**Table 3:** Partial correlations between Context Sensitivity Change scores and each of Listening (SAP), Language (GCC) and Attention (Cognitive Problems/Inattention), while controlling for the remaining two variables (italicised parentheses).

	Zero Order correlation	Partial correlation
	Context Sensitivity Change Score	Context Sensitivity Change Score
**Listening** *(Language & Attention)*	0.465, 0.003	−0.138, 0.417
**Attention** *(Language & Listening)*	−0.491, 0.002	−0.281, 0.093
**Language** *(Listening & Attention)*	0.614, 0.001	0.425, 0.009

## Discussion

This study assessed the hypothesis that parental perception that a child has listening difficulties is associated with weaknesses in the child’s ability to sustain attention while listening to speech over extended periods of time. To test this hypothesis, we used a task which bore some resemblance to a real world-listening situation. Our results suggest it is too simple to attribute symptoms of listening difficulty to a single deficit like attention. Instead, as we will argue, these symptoms reflect a complex inter-relationship between task demand and abilities across both cognitive and linguistic domains.

Initial results from the continuous listening task appeared to support the primary hypothesis that parental perception of listening difficulties associated with difficulties sustaining attention. Though children rated as having listening difficulties missed more mispronunciations than their counterparts with typical listening abilities, both groups were similarly sensitive to effects due to sentence context, with predictable targets being identified more reliably and more quickly than unpredictable targets. However, this initial sensitivity to sentence context faded over time in the children with listening difficulties, while a similar decline in detection of nonsense words was not observed. Further exploratory analyses suggested this decline in context sensitivity was primarily associated with underlying weaknesses in language abilities.

The finding of an increasing association with language weaknesses over time on listening ability is interesting in the context of earlier work by Dawes and Bishop^26^, who systematically compared the psychometric profiles of children diagnosed with either APD or dyslexia. They found the two groups were indistinguishable, apart from a discrepancy between parental report of language abilities and performance on standardised tests of language ability in the children diagnosed with APD. Essentially, the objectively measured language abilities of the children diagnosed with APD were better than parental report suggested. Dawes and Bishop proposed a role for communication demands in influencing the degree to which language difficulties are observable in these children. Our findings provide evidence in support of this hypothesis. Task demand clearly plays an important role in determining whether, or how, symptoms of listening difficulty manifest. It is possible that what distinguishes children referred with suspected APD, from those referred for suspected language difficulties, is that their weaknesses in language processing only become apparent over extended periods of listening. This, in turn, may prove a contributing factor when making decisions regarding referral route.

There is considerable controversy about whether APD, which presents as a profile of listening difficulties, is a distinct disorder in its own right or whether it is another term for SLI^5,7^. Our findings argue against distinct diagnostic categories in favour of a single neurodevelopmental syndrome^27^, where task demand plays an important role in influencing the profile of presenting symptoms.

The findings also raise questions about how listening difficulties should be assessed in the clinic. Specifically, they highlight the importance and relevance of redirecting focus away from the unsupported clinical protocols that are currently used to assess auditory processing abilities, towards tests designed to assess everyday listening functionality^3,28^. Our findings further suggest task duration may be an important consideration for such tests.

Apart from offering insights into the nature of presenting symptoms associated with listening difficulty, this study also demonstrates the value of using parental report to support the assessment of children.

Parental report measures have been criticised for being open to responder bias^8^. Our own data suggest parents, wittingly or otherwise, can be reliable, sensitive observers. The LiD-NonRef group of children were identified using report-based measures as having clinically significant listening difficulties, yet their carers were not actively seeking help for them. The children subsequently proved to be indistinguishable from the LiD-Ref group on all the report-based measures in the study, as well as the continuous listening task. Similar sensitivity of report-based measures to clinically significant, but unacknowledged language difficulty, has also been noted in the context of SLI^29^. These parallel findings suggest psychometrically robust questionnaires have a valuable role to play, not only in the assessment of children with recognized difficulties but also, in helping to identify children in need of support.

Gathercole *et al*.^30^ have previously noted an association between parental report of symptoms of inattention and working memory deficits. These difficulties are also frequently reported to characterise children with language^24,31^ or listening difficulties^17,32^. We did not demonstrate a clear relationship between our derived Context Sensitivity Change score and working memory, as assessed using the digit span backwards task. This suggests associations between poor working memory and symptoms of inattention may be circumstantial rather than causal. This suggestion would need to be further assessed with different measures of working memory.

### Study limitations

The report-based measures used in this study were chosen to capture apparently different aspects of cognitive or linguistic function. Nonetheless, these apparently different measures correlate quite highly, suggesting they are tapping into similar latent traits. In part, this reflects a general limitation of report-based measures – they capture symptoms, not causes. A parent cannot tell whether a blank look reflects problems with hearing, language, memory or attention.

The continuous listening task was designed to provide insight into the role of attention when listening to connected speech over extended periods of time. However, it is still an artificial task based on a complex, albeit more natural, stimulus. Because of the complexity of the stimulus, we cannot exclude effects on target detection from a host of factors influencing detectability, including acoustic, phonological and contextual effects. Nonsense words were included to address some of these problems. Unfortunately, although children understood the nature of a ‘silly word’ when presented out of context, they were less reliable at detecting these target types in context. The nonsense words were phonotactically legal and received morphological inflections as appropriate to the sentence context. This may been encouraged children to perceive them as ‘new’ words that they did not know, rather than as ‘nonsense’ words to be detected^33^. Regardless, these observations underline how use of context, while providing insight into on-line language processing, also complicates outcomes and interpretation.

## Conclusion

Previous evidence for an association between listening difficulties and sustained attention has come from artificial continuous performance tests, or psychophysical tasks of auditory processing abilities. Here, we showed how problems sustaining attention are also apparent in tasks involving connected speech, which more closely resemble natural listening. However, rather than a simple association between ability to sustain attention and report-based measures of listening or attention, the results suggest a complex inter-relationship, whereby strengths or weaknesses in the linguistic domain interact with capacity to sustain attention. In the absence of clearly identifiable problems with language, parents may attribute these effects to underlying listening difficulties.

## Methods

This study was approved by the Nottingham Research Ethics Committee 1. Informed consent was received from all participants and procedures complied with the British Psychological Society Code of Ethics and Conduct.

### Participants

To participate in the study, participants had to be native speakers of English, have normal hearing (pure tone hearing thresholds of 25 dB HL or better for frequencies: 250, 500, 1000, 2000 and 4000 Hz), a non-verbal IQ (NVIQ) ≥80 (WASI)^34^ and no pre-existing diagnosis of ADHD. This latter information was obtained using a questionnaire designed to establish a clinical case history, where parents provided information about suspected or diagnosed ADHD, dyslexia, SLI, or autism spectrum disorder.

Fifty-two participants, aged 7–13 years, were recruited from local schools (n = 32) or from audiology clinics (n = 20; LiD-Ref) in the East Midlands area of the UK. Eight participants (5 from local schools) were subsequently excluded, either because of missing data, or for not meeting recruitment criteria.

Children recruited from local schools were designated LiD or TLi based on parental responses on the Speech & Auditory Processing (SAP) subscale of the Evaluation of Children’s Listening and Processing Skills (ECLiPS; described below).

Twenty children were identified as having typical listening abilities (TLi). Seven children, however, had standard scores <7 on the ECLiPS: SAP subscale. Their difficulties were relevant to the study question, but they did not have a clinical referral for suspected APD. They were, therefore, designated ‘LiD-NonRef’, and initially kept separate from the children with a clinical referral.

Table 1 summarises data describing the participants. In addition to symptoms of listening difficulty, parental report-based measures suggest more problems with language and attention in the two LiD groups. The two groups are indistinguishable from each other, but statistically significantly different to the TLi group. Only one child in the LiD-NonRef group had an additional diagnosed comorbidity: dyslexia. This contrasted with the LiD-Ref group, where six children had additional diagnoses. Four children had a diagnosis of dyslexia, one had a diagnosis of reading and language delays, and one child had a diagnosis of dyspraxia. None of the children in the TLi group had any diagnosed difficulties.

### Screening questionnaires

In addition to the clinical case history questionnaire, three questionnaires were used to screen for difficulties with listening, language and attention.

The Evaluation of Children’s Listening and Processing Skills (ECLiPS)^12^ looks at listening in the context of language and social abilities and comprises 37 statements forming 5 subscales (*Speech & Auditory Processing* (SAP); *Language/Literacy/Laterality* (LLL); *Pragmatic & Social Skills*; *Environmental & Auditory Sensitivity*; *Memory & Attention*). Standard scores ≤6 are considered to be clinically significant. Reflecting the interests of the study, data from two subscales only are reported: *Speech & Auditory Processing* (SAP) and *Language/Literacy/Laterality* (LLL).

The Children’s Communication Checklist (CCC-2)^35^ screens for structural and pragmatic language difficulty. It comprises 70 items forming 10 subscales from which two composite measures are derived. We report the general communication composite (GCC), where scores <55 indicate clinically significant difficulty with language.

The Conners’ Parent Rating Scales-Revised Short Form (CPRS-R:S)^36^ screens for attention deficits in three domains (cognitive problems/inattention; hyperactivity and opposition). Since our hypothesis was specific to symptoms of inattention, we report scores from the cognitive problems/inattention subscale only. T-scores >64 on this domain indicate clinically significant difficulty.

### Tasks

#### The Continuous Listening Task – Jamie’s Story

The continuous listening task, “Jamie’s story” comprised a 2550 word story lasting 16 minutes with 108 targets (either 36 nonsense words or 36 × 2 mispronunciations) embedded within it. The target words were spaced between 6–94 words (4–32 seconds) apart. An excerpt of the story is provided in Appendix 1.

The 36 mispronunciations involved changing a single consonant at the beginning, or in the middle of a word that would be familiar to the children e.g. ‘paper’ to ‘**d**aper’. These words were presented in two different contexts. In one context, the target could be predicted from the preceding information in the sentence, for example, ‘He was reading the morning **d**aper’, while in the other, it could not, for example, ‘All she could find was a boring **d**aper’.

The 36 nonsense targets, for example, ‘tegwops’, were selected from the Test of Word Reading Efficiency^37^. They were phonologically legal, but did not sound like possible variants of known words.

The nonsense words were individually matched with the mispronunciations for syllable number (between one and three) and part of speech (noun, verb, adjective). Additional sentences, based on the vocabulary of the story were inserted to accommodate them and appropriate morphological inflections were added as required.

The story was subdivided into two halves, and the three target types were distributed equally across each half, with 18 nonsense words and 36 mispronunciations (18 predictable and 18 unpredictable) per half. Targets that were predictable in the first half of the story were not in the second, and vice versa.

The continuous listening task was presented using Matlab. Participants were seated in front of a laptop and a button box, with their hand held lightly on the button ready to respond as quickly as possible. The story was presented diotically over sound-attenuated Sennheiser HD 25-1 headphones at a comfortable listening level of 65 dB SPL. No visual information was provided during listening.

First the children were familiarised with the task requirements. As part of this, the concept of a ‘silly’ word was explained. They were told that some words, like ‘flibble’, would not mean anything at all, while others would sound like words that been said incorrectly. To illustrate, the tester said: “Eyes, Mouth, Dose… which is the silly word?”, while pointing to the relevant parts of her face. All participants immediately recognised the ‘silly word’.

Once familiarisation was complete, participants were instructed to listen carefully to the story and push the button as soon as they heard a silly word. They were also told they would be asked questions about the story at the end.

On conclusion of the task, the children were asked 8 questions regarding specific details from the story. They then completed a word recognition and word identification task (see below).

Numbers of targets detected and reaction times were recorded, for later extraction and analysis. Target detection was defined as a response occurring within a window between 250 ms to 3000 ms after target onset^25^.

#### Word Recognition Task

To ensure all participants were familiar with the words used to generate the target mispronunciations, they completed a word recognition task. The unmodified words were presented over headphones using Psychopy^38^ together with a choice of either four, or in two instances one, coloured picture(s). The participants pointed to the picture (or feature in the picture) corresponding to the word they had heard.

#### Target Identification Task

A target identification task was used to verify that all participants could perceive the mispronunciations when presented in isolation. The 36 mispronunciations and an additional 36 words from the story, individually matched for syllable length and part of speech to a mispronunciation, were presented in a two-alternative forced-choice task (Psychopy)^38^. Participants had to indicate whether they thought the word was ‘silly’ or real. If a participant missed a mispronunciation in both this task and the story task, it was excluded from further analysis.

#### Digit span backwards

Deficits in working memory are often associated with difficulties with language^31,39^, and listening^32^, as well as with symptoms of inattention^30^. A measure of working memory capacity – the digit span backward task (WISC-IV)^40^ – was therefore obtained.

Participants listened to strings of digits presented over headphones and repeated them in reverse order. Two trials per string length were presented and the test stopped when the participant failed two trials at a particular length. The number of trials correctly repeated were summed and converted into standard scores.

### Recording of task stimuli

All speech materials were recorded in a soundproof booth with a trained male speaker of standard British English. The recordings were made using a Tascam USB Audio Interface with Behringer B-2 Pro microphone and digitised in Goldwave (16 bit-depth, 44.1 kHz sampling rate).

Individual words and targets for the word recognition and identification tasks were recorded three times using a short carrier phrase. This was excised and the best exemplar of each word and target was retained.

When recording the story, the speaker was instructed to read it at a comfortable speaking rate. All targets (mispronunciation and nonsense) were practiced in isolation before three separate recordings of the story were made to obtain a clear, artefact-free version for use in testing.

The level for all stimuli was root mean square equalised in Audacity.

### Procedure

All participants completed a large test battery. In addition to assessment of NViQ and the continuous listening task, the battery included different CPTs, tests of short-term memory, and a test of speech-in-noise perception. To minimise effects due to fatigue, testing was split into two sessions of seventy-five minutes each. Additional breaks were provided as required. Testing began with pure tone audiometry. The order of the remaining tests was pseudo-randomised and counterbalanced across participants. There were two key requirements for the test protocol. First, no more than two CPTs were permitted per test session, and these tasks never directly followed each other. Secondly, the story task was always followed by the questions about it, the word recognition task, and finally the word identification task, in that order.

### Statistical analyses

Results are presented as percent missed targets (total and per type). Percentages intrinsically lack a normal sampling distribution, moreover Kolmogorov-Smirnov tests for mispronunciations were significant (p < 0.05). Prior to analysis using parametric tests (ANOVA), target detection percentages and derived subtraction variables (errors (half1 – half2)) were transformed using a rationalised arcsine transformation^41^. Back-transformed values are reported in the text. Greenhouse-Geisser corrections address violations of sphericity and are reported as necessary. Kolmogorov-Smirnov tests for reaction time did not significantly deviate from normality (p > 0.05), transformations were not required prior to analysis. Multiple comparisons for the ANOVA’s were corrected with Bonferroni adjustment. Multiple comparisons for correlations were Holm-Bonferroni corrected. All analyses were performed in SPSS (v.21).

## Electronic supplementary material

Appendix 1
