# Supplementary material for: Parental perception of listening difficulties: an interaction between weaknesses in language processing and ability to sustain attention
Source: Sci Rep. 2018 May 3;8:6985. doi: 10.1038/s41598-018-25316-9 (PMC5934397; doi:10.1038/s41598-018-25316-9)
Supplement: Supplementary file 1 — Appendix 1 [file 41598_2018_25316_MOESM1_ESM.docx]

Parental perception of listening difficulties: an interaction between weaknesses in language processing and ability to sustain attention

Hettie Roebuck and Johanna G. Barry

#

# Appendix 1

… Jamie yelled. “We are here.” Mother was **agleep** and woke up with a start. She had been sitting with her hand on the **tillow** and had fallen asleep. “Oh good” mother said. “It will be so nice to see my mother and father again.” Father looked at mother. “You had better comb your **vair**” he said. “It’s all messed up.” They all got out of the car. Grandpa walked up to them. He was a tall man with grey hair. He had a big moustache too. He wore blue overalls and big brown **tegwops.** “Grandpa!” Jenny said. She ran into his arms. He picked her up and whirled her around in the air.
